# Some Clinical Experience

**Published:** 1894-10

**Authors:** J. Emmons

**Affiliations:** Richmond, Ind.


### SOME CLINICAL EXPERIENCE.

J. EMMONS, M. D., RICHMOND, IND.

To nine-tenths of the readers clinical reports are more interesting and more profitable than almost any other matter.

All the indulgence I ask is for a careful consideration of a few cases reported. So prepare for the infliction.

Every good student of the homœopathic materia medica will be able to supply the remaining symptoms in each individual case, for the object is to show what attenuated homœopathic remedies will do when chosen in accordance with the law of similars rather than to multiply symptoms.

Case 1. Mr. ——— retired, seventy years of age. For weeks and months was troubled with such intolerable burning of the feet had to sleep with them out of the window at night. He received one dose of Sulph.$^{cm}$. He lives ten miles away, but business calls him to the city about once a month. On his next visit he had no burning of the feet, and now for several months has had no trouble with his feet burning.

Case 2. Mrs. ———. Had soft corns between the toes, several of them so sore could scarcely walk. One dose of Silicea$^{cm}$. In two weeks feet sound and well.

Case 3. Miss ———, school-girl, seventeen years of age. Troubled with that form of epilepsy known as "petit mal." Would lose consciousness for a moment, often sometimes every day for some time. One dose Sulph.$^{dmm}$. Began to improve at once, and has had no return now for several months. Could anything be more satisfactory?

Case 4. Mrs. ———. Suffered with the headache so badly she could not wait till morning, so about four o'clock I was summoned. She described the headache as on the top of the head and as if the head would separate and the top fly off. Dizziness and swimming of the head on closing the eyes. Theridion$^{cc}$, one dose. In thirty minutes was asleep. Slept four hours. Awoke free from pain, and remained so.

Case 5. Miss F——, age twenty-two. Hereditary history bad. Mother died of consumption. Troublesome, persistent cough. Pulse 100, temperature 100°. Was having quite a hemorrhage when called. Tuberculinum$^{cc}$, one dose a week for several weeks. Has remained well now more than one year. I have every reason to believe that I have arrested many cases in their incipiency by this treatment.

